# Association Analysis of the Genomic and Functional Characteristics of Halotolerant *Glutamicibacter endophyticus* J2-5-19 from the Rhizosphere of *Suaeda salsa*

**DOI:** 10.3390/microorganisms13010208

**Published:** 2025-01-18

**Authors:** Longhao Sun, Shanshan Sun, Tianyang Liu, Xinmin Lei, Ruiqi Liu, Junyi Zhang, Shanshan Dai, Jing Li, Yanqin Ding

**Affiliations:** 1 Department of Microbiology, College of Life Sciences, Shandong Agricultural University, Taian 271018, China; 2 Shandong Engineering Research Center of Plant-Microbial Restoration for Saline-Alkali Land, Taian 271018, China

**Keywords:** saline-alkali soil, Halotolerant plant growth-promoting bacteria, *Glutamicibacter endophyticus*, Genomic analysis

## Abstract

Halotolerant plant growth-promoting bacteria (HT-PGPB) have attracted considerable attention for their significant potential in mitigating salt stress in crops. However, the current exploration and development of HT-PGPB remain insufficient to meet the increasing demands of agriculture. In this study, an HT-PGPB isolated from coastal saline-alkali soil in the Yellow River Delta was identified as *Glutamicibacter endophyticus* J2-5-19. The strain was capable of growing in media with up to 13% NaCl and producing proteases, siderophores, and the plant hormone IAA. Under 4‰ salt stress, inoculation with strain J2-5-19 significantly increased the wheat seed germination rate from 37.5% to 95%, enhanced the dry weight of maize seedlings by 41.92%, and notably improved the development of maize root systems. Moreover, this work presented the first whole-genome of *Glutamicibacter endophyticus*, revealing that *G. endophyticus* J2-5-19 resisted salt stress by expelling sodium ions and taking up potassium ions through Na^+^/H^+^ antiporters and potassium uptake proteins, while also accumulating compatible solutes such as betaine, proline, and trehalose. Additionally, the genome contained multiple key plant growth-promoting genes, including those involved in IAA biosynthesis, siderophore production, and GABA synthesis. The findings provide a theoretical foundation and microbial resources for the development of specialized microbial inoculants for saline-alkali soils.

## 1. Introduction

Soil salinization is a major agricultural issue, as salt stress can negatively impact the entire lifecycle of plants, greatly limiting the productivity of arable land. Statistics show that about 1 billion hectares of land globally are affected by salinization [1].

Halophytes are the dominant species in saline-alkali soils, and plant growth-promoting bacteria (PGPB) present in the roots and rhizosphere of halophytes help support their resistance and adaptation to salt stress [2]. Compared to plant growth-promoting bacteria in non-saline soils, those associated with halophytes generally exhibit greater tolerance to high salt concentrations [3]. These bacteria are referred to as Halotolerant plant growth-promoting bacteria (HT-PGPB). HT-PGPBs have significant potential for alleviating salt stress in non-halophyte plants, and in recent years, numerous studies have highlighted their application in mitigating crop salt stress. These strains are distributed across 21 genera, including *Bacillus*, *Pseudomonas*, *Arthrobacter*, *Kocuria*, *Serratia*, and others (Table 1).

*Glutamicibacter endophyticus* was originally isolated from the roots of the halophyte *Salsola affinis* C. A. Mey in the Urumqi area of Xinjiang, China. It was identified as a new member of the genus *Arthrobacter*, and was named *Arthrobacter endophyticus*, with the type strain EGI 6500322T (=DSM 28750=JCM 30091=KCTC 29490) [4]. Busse reviewed the taxonomic studies of the genus *Arthrobacter* and proposed *Glutamicibacter* as a new taxonomic unit [5]. He also conducted a re-analysis of the peptidoglycan structure and polar lipid profile of *A. endophyticus*, which led to its reclassification as *Glutamicibacter endophyticus* [6].

Existing research has shown that *Glutamicibacter endophyticus* is a species closely associated with plants. Sadeghi et al. isolated a strain of *Glutamicibacter* sp. N1A3101, which is closely related to *G. endophyticus* EGI 6500322T, from soil samples of the roots of *Urtica dioica* (nettle) in Iran. This strain is capable of producing heat-stable alkaline histamine oxidase [7]. Khan et al. isolated *G. endophyticus* TKE2, TKR6, and TKR8 from both the roots and rhizosphere soil of the halophyte *Arthrocnemum macrostachyum* in Karachi, Pakistan. These strains have the ability to secrete various extracellular enzymes [8]. Dong et al. reported an endophytic strain *G. endophyticus* SYSU 333322 isolated from the halophyte *Borszczowia aralocaspica* in the Shihezi area of Xinjiang, China, which can enhance the resistance of *Arabidopsis thaliana* to salt and alkali stress [9].

The salt tolerance and plant growth-promoting effects of this species on crops, as well as its genomic characteristics, have not been reported. We isolated a highly salt-tolerant bacterium, J2-5-19, from the rhizosphere soil of *Suaeda salsa*, a halophyte, in the Yellow River Delta region. The salt tolerance and plant growth-promoting functions of strain J2-5-19 were investigated, and it was identified as *Glutamicibacter endophyticus* based on phenotypic traits and 16S rRNA gene phylogenetic analysis. *G. endophyticus* J2-5-19 exhibits plant growth-promoting abilities, including the secretion of protease, iron siderophores, and indole-3-acetic acid (IAA). Further experiments showed that *G. endophyticus* J2-5-19 significantly increased the germination rate of wheat seeds and promoted the growth of maize under salt stress. To better understand this strain, we sequenced the complete genome of *G. endophyticus* J2-5-19 and investigated its salt tolerance and plant growth-promoting mechanisms at the genomic level.

**Table 1:** Application of Salt-Tolerant PGPBs in Crops.

Bacterial Species	Source Halophyte	Applied Crops	References
*Agrobacierium tumefaciens* *Klebsiella* sp. *Ochrobactrum anthropi* *Pseudomonas stutzeri* *Pseudomonas* sp.	*Arthrocnemum indicum*	peanut	[10]
*Arthrobacter woluwensis* AK1 *Microbacterium oxydans* AK2 *Arthrobacter aurescens* AK3 *Bacillus megaterium* AK4 *Bacillus aryabhattai* AK5	*Artemisia princeps* *Chenopodium ficifolium* *Oenothera biennis* *Echinochloa crus-galli*	soybean	[11]
*Bacillus* sp. *Arthrobacter pascens*	*Atriplex leucoclada* *Suaeda fruticosa*	maize	[12]
*Bacillus pumilus* HR *Zhihengliuella halotolerans* SB	*Seidlitzia rosmarinus* *Halostachys belangeriana*	wheat	[13]
*Bacillus pumilus* STR2 *Halomonas desiderata* STR8 *Exiguobacterium oxidotolerans* STR36	*Poaceae*	*Mentha arvensis*	[14]
*Bacillus inaquosorum* *Bacillus thuringiensis* *Bacillus proteolyticus*	*Limoniastrum monopetalum* *Arthrocnemum indicum* *Holocnemum strobilaceum*	quinoa (*Chenopodium quinoa*)	[15]
*Enterobacter asburiae* A103	*Salix linearistipularis*	*Medicago sativa*	[16]
*Glutamicibacter halophytocola* KLBMP 5180	*Limonium sinense*	tomato	[17]
*Kocuria turfanensis* 2M4	*Suaeda fruticosa*	peanut	[18]
*Pseudarthrobacter oxydans* B9 *Staphylococcus pasteuri* B10	*sorghum*	tomato	[19]
*Paraburkholderia* sp. GD17	*Glycine soja*	rice	[20]
*Pseudomonas putida* CO1 *Bacillus paramycoides* CO8	*Cocos nucifera*	French bean (*Phaseolus vulgaris*)	[21]
*Pseudomonas* sp. TE7 *Providencia rettgeri* SE5 *Pantoea agglomerans* SE19	*Tamarix gallica* *Suaeda fruticosa*	barley tomato	[22]
*Pseudomonas chloritidismutans* 6L11	*Salicornia*	wheat	[23]
*Streptomyces* sp. KLBMP5084	*Limonium sinense*	tomato	[24]
*Serratia marcescens* *Bacillus velezensis* *Kocuria rhizophila* *Kosakonia radicincitans*	*Sesuvium portulacastrum*	*Vigna mungo*	[25]
*Serratia* sp. NTN6	*Puccinellia tenuiflora*	maize	[26]
*Stenotrophomonas maltophilia* BJ01	*Cyperus laevigatus*	peanut	[27]
*Variovorax* sp. P1R9	*Distichlis spicata*	wheat	[28]

## 2. Materials and Methods

### 2.1. Soil Sample Collection and Strain Isolation

Rhizosphere soil samples of *Suaeda salsa* were collected from an oil drilling site in Dongying City, Shandong Province, China (37°24′21.50″ N, 118°29′46.00″ E). The sampling site is located in the Yellow River Delta, where the soil is classified as coastal saline soil, with a salt composition similar to seawater, predominantly composed of chloride salts (China Soil Database).

The soil enveloping the root systems of *Suaeda salsa* plants was dug up with a shovel, placed in sterile sealed bags, and transported to the laboratory at low temperature within 8 h. In a laminar flow hood, the bulk soil was gently shaken off to expose the roots, and the rhizosphere soil adhering closely to the roots was collected using a sterile brush. A total of 5 g of rhizosphere soil was inoculated into Sehgal–Gibbons (S-G) liquid medium containing 5% NaCl, which is composed of 7.5 g/L casein acid hydrolysate, 10 g/L yeast extract, 3 g/L trisodium citrate dihydrate, 20 g/L MgSO_4_·7H_2_O, 2 g/L KCl, 0.05 g/L FeSO_4_·7H_2_O, and 50 g/L NaCl, with a pH of 7.0–7.2. The culture was incubated at 28 °C in a shaking incubator at 180 rpm for 5 days [29]. The resulting suspension was serially diluted to 10^−6^, and 100 µL aliquots of the 10^−4^, 10^−5^, and 10^−6^ dilutions were plated onto 5% NaCl S-G agar plates. Single colonies were isolated and repeatedly purified to obtain a pure culture. The strain J2-5-19 has been deposited at the China Center for Type Culture Collection (CCTCC) with the accession number CCTCC M 20241774.

The bulk soil was air-dried, and extracts were prepared using deionized water to measure pH (1:2.5, *w*:*v*) and electrical conductivity (EC) (1:5, *w*:*v*). The soil extracts had a pH of 7.81 and an EC of 23.2 mS/cm, indicating that the sample is from highly salinized soil.

### 2.2. Strain Identification

#### 2.2.1. Morphological Identification

The strain was cultured on Luria–Bertani (LB) medium, and its colony morphology was recorded. Gram staining was performed using a Gram stain kit (SL7040, Coolaber, Beijing, China) following the manufacturer’s standard protocol, and the stained cells were observed under an optical microscope (BX53, OLYMPUS, Tokyo, Japan). Bacterial cells from overnight cultures in the LB liquid medium were collected by centrifugation. The cell pellet was then resuspended in a 2.5% glutaraldehyde solution and fixed for 3 h. After fixation, the cells were washed with PBS buffer, followed by gradient dehydration using ethanol solutions of 30%, 50%, 70%, 80%, 90%, and 100%. After dehydration, tert-butanol was used to replace ethanol in the samples three times, and the samples were finally freeze-dried to remove the tert-butanol. The cell morphology was observed under a field emission scanning electron microscope (GeminiSEM 300, ZEISS, Oberkochen, Germany) after platinum sputter coating.

#### 2.2.2. Biochemical Identification

The biochemical characteristics of the strain were identified using the API 20NE kit (bioMérieux, Marcy-l’Étoile, France). The MR-VP test and starch hydrolysis test were performed using commercial identification media (Hopebio, Qingdao, China). The hydrogen sulfide test, Tween hydrolysis test, and oxidase test were carried out according to standard bacterial identification manuals [30]. All experiments were performed in triplicate.

#### 2.2.3. 16S rRNA Gene Identification

The 16S rRNA gene was amplified using the primers 27F (5′-AGAGTTTGATCCTGGCTCAG-3′) and 1492R (5′-TACGACTTAACCCCAATCGC-3′) from fresh colony. The PCR reaction volume was 50 μL, consisting of 4 μL of primer mix (2 μL of each primer), 25 μL of 2× PCR reaction mix (containing DNA polymerase, dNTPs, buffer, and other necessary components), and 19 μL of ultrapure water. PCR conditions were as follows: initial denaturation at 95 °C for 3 min, followed by 31 cycles of 30 s at 94 °C, 30 s at 55 °C, and 1 min 30 s at 72 °C, with a final extension at 72 °C for 5 min. The PCR products were sent to Sangon Biotech for Sanger sequencing.

The sequences were initially analyzed using the blastn tool from NCBI. Type strain 16S rRNA gene sequences of the *Glutamicibacter* genus were obtained from LPSN (https://lpsn.dsmz.de/) (accessed on 18 December 2024) as reference sequences. A phylogenetic tree was constructed using MEGA 7.0 software.

### 2.3. Functional Characterization and Plant Growth Promotion Experiments

#### 2.3.1. Salt Tolerance Evaluation of Strains

Seed cultures with an OD600 of 1.0 were prepared and inoculated into an LB liquid medium containing NaCl at concentrations ranging from 0 to 15% (inoculum size: 1%). The cultures were incubated at 37 °C and 180 rpm in a shaking incubator. Samples were collected every 6 h to monitor strain growth.

#### 2.3.2. Evaluation of Plant Growth-Promoting Abilities

The protease production ability of the strains was assessed using casein agar containing bromocresol blue (Hopebio, Qingdao, China) as an indicator. The siderophore production ability of the strains was evaluated using CAS plates [31].

Strain J2-5-19 was inoculated into LB medium and LB medium supplemented with 5 mM tryptophan. The cultures were incubated at 28 °C and 180 rpm in a shaking incubator for 48 h. The fermentation broth concentration (OD_600_) was measured. After centrifugation, the cell-free supernatant was mixed with an equal volume of Salkowski reagent (1 mL of 0.5 M FeCl_3_ dissolved in 50 mL of 35% perchloric acid). After a 30-min reaction in the dark, the OD_530_ value was measured. The standard curve was used to calculate the IAA concentration in the cell-free supernatant [32]. The IAA unit yield in the fermentation broth was determined by calculating the ratio of the IAA concentration in the cell-free supernatant to the fermentation broth concentration (OD_600_).

For IAA standard curve preparation, standard solutions of IAA at concentrations of 0, 5, 10, 20, 50, and 100 μg/mL were prepared using LB as the solvent. An equal volume of Salkowski reagent was added to each standard solution. After a 30-min reaction at 37 °C in the dark, the OD_530_ was measured. The standard curve was plotted with OD_530_ on the *x*-axis and the standard solution concentration on the *y*-axis: y = 34.838x − 2.2626 (R^2^ = 0.9962).

#### 2.3.3. Germination Experiment of Wheat Seeds Under Salt Stress

Salt stress conditions simulating coastal saline-alkali soil were created using sea salt (major components: NaCl 92%, Ca^2+^ 0.32%, Mg^2+^ 0.22%). Fresh bacterial cells were collected from the LB medium by centrifugation to prepare suspensions at concentrations of 10^8^, 10^7^, and 10^6^ cfu/mL. Wheat seeds (variety: Jimai 22) were soaked in these suspensions, while the control group seeds were treated with an equal volume of sterile water. The germination experiment under salt stress was conducted using quartz sand as the substrate, which was saturated with a 4‰ sea salt solution. The seeds were cultured at 25 °C for 7 days with 16 h of light and 8 h of darkness per day. Every day, a 4‰ sea salt solution was sprayed onto the substrate to maintain its maximum water-holding capacity, ensuring a constant salinity level throughout the cultivation process. Three replicates were set for each treatment.

#### 2.3.4. Corn Pot Experiment Under Salt Stress

The pot experiment was conducted in a greenhouse at Shandong Agricultural University. The test soil was air-dried agricultural soil (with a salt content of 0.46‰), and 2 kg of soil was placed in each pot. After sowing the corn seeds (variety: Zhengdan 958), the plants were allowed to grow until the two-leaf and one-heart stage, with uniformly healthy seedlings selected for the experiment.

The LB culture containing 10^9^ viable bacteria was diluted with 400 mL of 2% sea salt solution, and 400 mL of the resulting suspension was applied to each pot. The control group was treated with an equal volume of sterile LB medium. Eight replicates were used for each treatment.

The aboveground height was measured after 30 days of treatment. The plants were then harvested, and root images were captured using EPSON GT-X980 scanner (EPSON, Suwa, Japan). The root images were analyzed using the WinRHIZO root analysis system (v2017C). Fresh weight was recorded, and after drying the plants in an oven at 105 °C until a constant weight was achieved, dry weight was measured.

### 2.4. Genome Sequencing and Analysis

#### 2.4.1. Genome Extraction

Bacterial cells in the logarithmic growth phase were collected by centrifugation, and genomic DNA was extracted using the CTAB method.

#### 2.4.2. Genome Sequencing and Assembly

Whole-genome sequencing was conducted at Personalbio (Shanghai, China) using the Illumina NovaSeq XPlus platform (Illumina, San Diego, CA, USA) and the PacBio Sequel II platform (PacBio, Menlo Park, CA, USA). The sequencing data were assembled into contig sequences using Unicycler (v0.5.0) and Flye software (v2.9.1). These contigs were then refined with second-generation sequencing data using Pilon software (v1.18) to obtain the complete genome sequence.

Gene prediction was performed using GeneMarkS software (v4.32). Non-coding RNA genes were predicted using tRNAscan-SE (v1.3.1), Barrnap (v0.9), and the Rfam database (v14.1). The CRISPR-Cas system was identified using CRISPRCasFinder (v4.2.20). Bacteriophages were predicted using PhiSpy (v4.2.21), and genomic islands were identified using IslandViewer 4.

#### 2.4.3. Comparative Genome Analysis 

Genome Blast Distance Phylogeny (GBDP) analysis was conducted using the Type (Strain) Genome Server (TYGS) (https://tygs.dsmz.de) (accessed on 18 December 2024). Genomes of type strains of *Glutamicibacter* species for comparative genomic analysis were obtained from the NCBI GenBank database and the GCM type strains genome database (Table S1). Digital DNA–DNA Hybridization (dDDH) was determined using the Genome-to-Genome Distance Calculator 3.0 [33]. Average Nucleotide Identity (ANI) was calculated using the JspeciesWS online tool (http://jspecies.ribohost.com/jspeciesws) (accessed on 20 September 2024).

#### 2.4.4. Gene Function Annotation

Genes encoding protein sequences were compared against the eggNOG (COG), NCBI NR, and UniProtKB Swiss-Prot databases using DIAMOND blastp (v2.0.11), with an E-value threshold of 1e-6.The best-hit COG identifier was assigned to each corresponding gene and visualized on a genomic circular map using different colors to represent their classifications. Secondary metabolite biosynthetic gene clusters in strain J2-5-19 were predicted using the antiSMASH 7.0 server (https://antismash.secondarymetabolites.org/) (accessed on 11 November 2024). Protein structure prediction was carried out using AlphaFold2 (v2.3.2), and the predicted structure was visualized using PyMOL (open source v3.0.0).

### 2.5. Statistical Analysis

The Shapiro–Wilk test was used to assess the normality of the data. For comparisons among multiple groups, the Kruskal–Wallis H test was applied to evaluate whether there were significant differences in the distributions across groups. When significant differences were identified, post hoc pairwise comparisons were performed using the Bonferroni correction. For comparisons between two independent groups, an independent samples *t*-test was used to assess whether there were significant differences between the means of the two groups. All statistical analyses were conducted using IBM SPSS Statistics 26 software, and statistical significance was determined based on the *p* value. All graphs were created using GraphPad Prism 8 software.

## 3. Results and Discussion

### 3.1. Morphological Characteristics of the Strain

**Figure 1:**
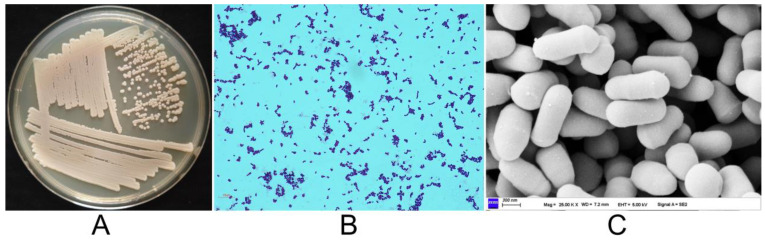
(**A**) Colony morphology of strain J2-5-19 grown on an LB agar plate. (**B**) Gram-staining of strain J2-5-19 observed at 1000× magnification; scale bar = 10 μm. (**C**) Cellular morphology of strain J2-5-19 observed by SEM at 25,000× magnification; scale bar = 300 nm.

Strain J2-5-19 grew well on S-G agar plates containing 5% NaCl as well as on LB agar plates without NaCl. The growth of this strain is not dependent on high salt concentrations, indicating that it is a salt-tolerant bacterium. On LB agar plates, strain J2-5-19 forms creamy, raised colonies (Figure 1A) with a smooth, moist surface. The strain was Gram-staining-positive and did not form spores (Figure 1B). The fresh samples cultured for 12 h exhibited rod-shaped cells with curved ends, measuring 0.5 µm × 0.8–1.6 µm under scanning electron microscopy (Figure 1C).

### 3.2. 16S rRNA Gene Sequencing

**Figure 2:**
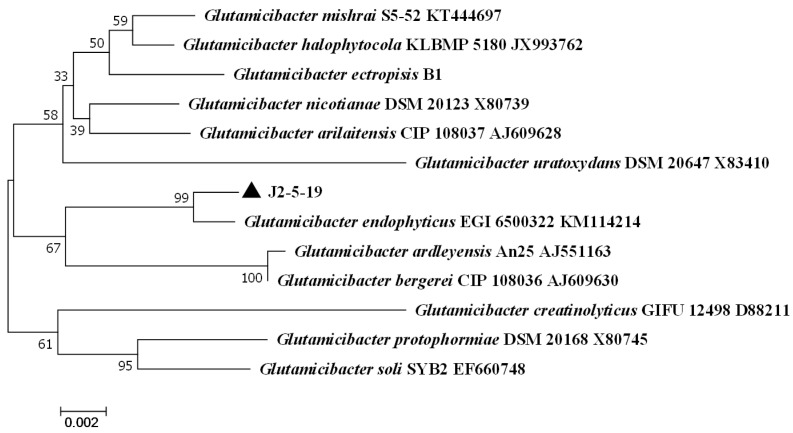
Neighbor-joining phylogenetic tree based on 16S rRNA gene sequences of strain J2-5-19 and representative *Glutamicibacter* species. Bootstrap values at each branch node indicate the percentage of support based on 1000 resampling replicates. Strain J2-5-19 clusters with *G. endophyticus* EGI 6500322T within the same clade. The scale bar represents 0.002 nucleotide substitutions per site.

Sequence comparison of 16S rRNA genes revealed that strain J2-5-19 shared the highest sequence similarity (99.57%) to *Glutamicibacter endophyticus* based on the 16S rRNA gene. A phylogenetic tree was constructed using the 16S rRNA gene sequences of all species within the Glutamicibacter genus as references, employing the neighbor-joining method. Strain J2-5-19 clustered with the type strain Glutamicibacter endophyticus EGI 6500322T in the same clade (Figure 2), with a bootstrap value of 100%. This phylogenetic classification was further confirmed by a tree constructed using the maximum likelihood method (Figure S1). Therefore, the isolate was preliminarily identified as *G. endophyticus* J2-5-19.

### 3.3. Biochemical Characteristics

*G. endophyticus* J2-5-19 is oxidase-negative, catalase-positive, and does not produce hydrogen sulfide (H_2_S). It yields negative results for both the methyl red (MR) and Voges-Proskauer (VP) tests. This strain is positive for the hydrolysis of starch and casein, but negative for the hydrolysis of Tween 20, Tween 40, Tween 60, and Tween 80. Additionally, it is negative for nitrate reduction, indole production, glucose fermentation, arginine dihydrolase, and urease activities. The strain is positive for the hydrolysis of aesculin, gelatin, and 4-nitrophenyl-β-D-glucopyranoside (PNPG). In terms of carbohydrate and acid assimilation, it is positive for glucose, arabinose, mannose, N-acetylglucosamine, maltose, gluconate, malic acid, citrate, and phenylacetic acid but negative for mannitol, adipic acid, and capric acid. Compared with the type strain *G. endophyticus* EGI 6500322, strain J2-5-19 exhibited different results in the hydrolysis of Tween 60, urea, and 4-nitrophenyl-β-D-glucopyranoside, as well as in the mannitol assimilation test (Table 2).

### 3.4. Genomic Architecture and Comparative Genomic Analysis

**Figure 3:**
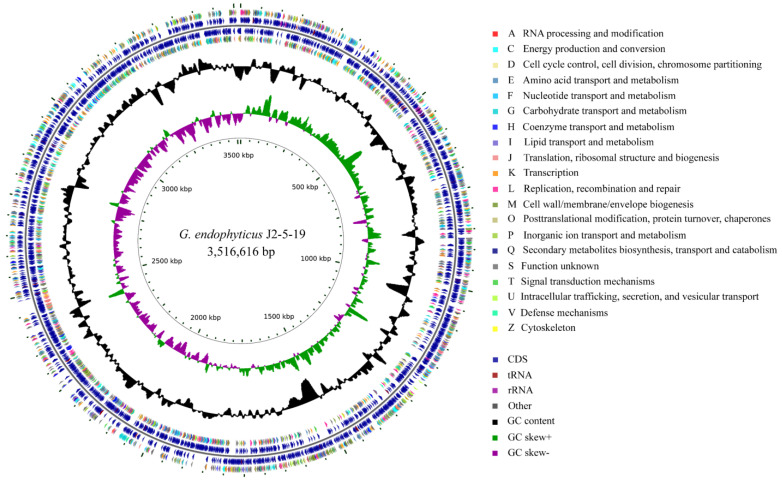
Genomic circle map of *G. endophyticus* J2-5-19. From inside to outside, the first circle represents the scale, the second circle represents GC skew, the third circle represents GC content, the fourth and seventh circles represent the COG to which each coding sequence (CDS) belongs, and the fifth and sixth circles represent the location of CDS, transfer RNA, and ribosomal RNA on the genome.

The complete genome of *G. endophyticus* J2-5-19 is a circular DNA molecule, with a total length of 3,516,616 bp and a GC content of 61.54% (Figure 3). It contains 3208 open reading frames, with an average length of 952.32 bp. The ORF region comprises 86.87% of the genome and has a GC content of 62.38%. A total of 2811 coding genes were annotated in the COG database and classified into 20 COG categories. The largest group, comprising 25.4%, consists of S-class genes with unknown functions. Genes from the following categories contribute over 5% of the total, including transcription (K) at 9.43%, amino acid transport and metabolism (E) at 8.93%, carbohydrate transport and metabolism (G) at 6.87%, inorganic ion transport and metabolism (P) at 6.37%, translation, ribosomal structure, and biogenesis (J) at 5.76%, replication, recombination, and repair (L) at 5.59%, and energy production and conversion (C) at 5.19%.

In the genome, six 5S rRNA genes, five 16S rRNA genes, five 23S rRNA genes, sixty-one tRNA genes, and twenty-eight other ncRNA genes were identified. Additionally, the genome contains two CRISPR-Cas system structures (Table S2), twenty-six genomic islands (Table S3), and twenty-five prophage regions (Table S4) were predicted.

**Figure 4:**
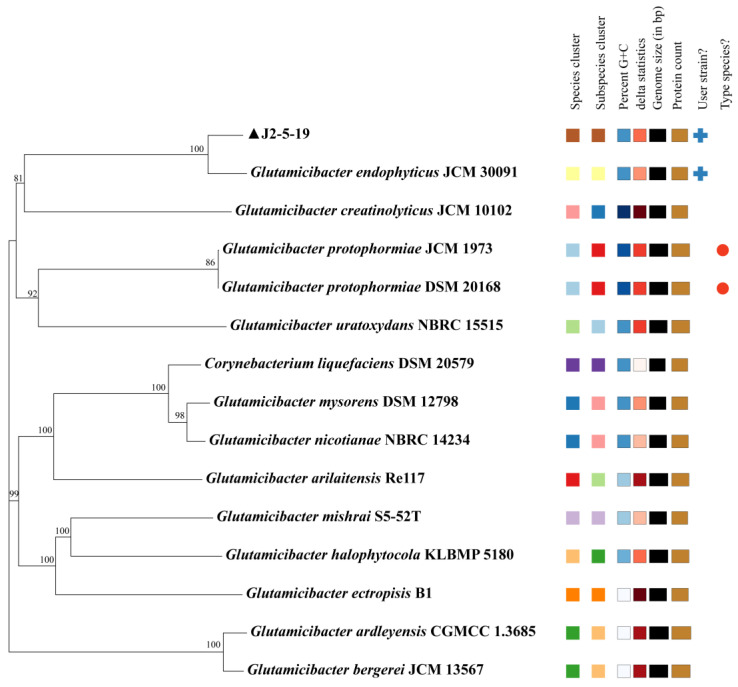
GBDP tree inferred with FastME 2.1.6.1 from GBDP distances calculated from genome sequences. The branch lengths are scaled in terms of GBDP distance formula d5. The numbers above branches are GBDP pseudo-bootstrap support values >60% from 100 replications, with an average branch support of 92.1%.

Using TYGS for Genome BLAST Distance Phylogeny (GBDP) analysis, the matched reference strains all belong to the *Glutamicibacter* genus. *G. endophyticus* J2-5-19 and *Glutamicibacter endophyticus* JCM 30091 cluster together on the phylogenetic tree, indicating a very close phylogenetic relationship between the two strains (Figure 4).

Considering that the reference genomes are draft sequences, we based our analysis on the digital DNA:DNA hybridization (dDDH) results calculated using Formula 2 (Identities/HSP length). The dDDH result indicates that the DDH estimate between J2-5-19 and *Glutamicibacter endophyticus* is 67.4% (64.4–70.3%), slightly below 70%. The GC difference between the two strains is only 0.01%. After performing Average Nucleotide Identity (ANI) calculations for species in the *Glutamicibacter* genus, we found that the ANIb and ANIm values between strain J2-5-19 and *Glutamicibacter endophyticus* were both above 95%. Based on the above analysis, strain J2-5-19 was identified as *Glutamicibacter endophyticus* (Table 3).

In addition, we found that the ANIb and ANIm values between *Glutamicibacter ardleyensis* and *Glutamicibacter bergerei* were both above 95%, and their DDH estimate was 79.7% (76.8–82.4%). The difference in GC content was 0.24%, indicating that the two strains are actually the same species. According to the principle of priority based on publication date, *Glutamicibacter ardleyensis* should be regarded as a synonym of *Glutamicibacter bergerei* [34,35].

### 3.5. Salt Tolerance and Mechanism of Salt Resistance in the Strain

**Figure 5:**
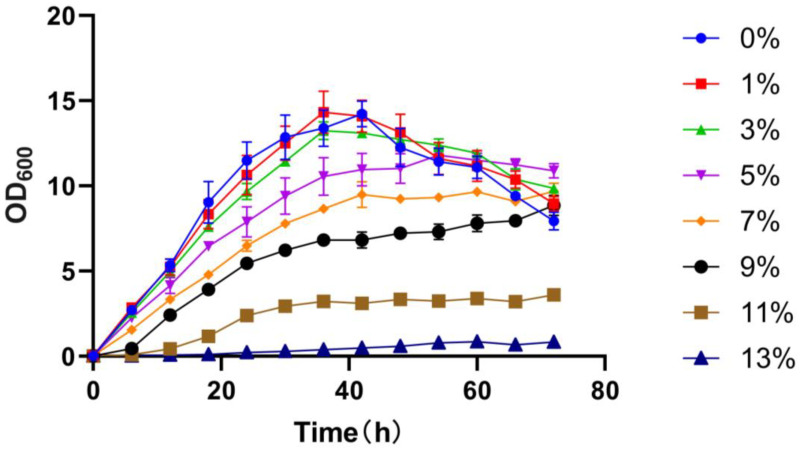
Growth curves of strain J2-5-19 under different NaCl concentrations. Data points represent the mean of three biological replicates (*n* = 3), with error bars indicating the standard deviation (SD) of the mean.

The salt tolerance experiment demonstrated that *G. endophyticus* J2-5-19 could grow within a NaCl concentration range of 0–13% (*w*/*v*). Over a 72-h cultivation period, the strain exhibited similar growth rates at NaCl concentrations of 0–3%, reaching maximum cell densities at around 40 h. Thereafter, the bacterial growth entered the decline phase (Figure 5). At 72 h, the cell densities in the media containing 5% and 7% NaCl were higher than those in 1% and 0% NaCl, with the strain remaining in a stationary phase. When the NaCl concentration exceeded 3%, bacterial growth was increasingly inhibited, with the degree of inhibition rising alongside the salt concentration. At a NaCl concentration of 13%, bacterial growth was minimal, with OD_600_ values remaining below 1.

In the genome of *G. endophyticus* J2-5-19, nine genes associated with Na^+^ efflux were annotated (Table S5). These included three genes encoding single-subunit Na^+^/H^+^ antiporters (*apnhaP*, *nhaA*, and *gerT*), and an *mrp* operon that encodes multiple resistance and pH adaptation (Mrp) cation/proton antiporters [36,37,38].

**Figure 6:**
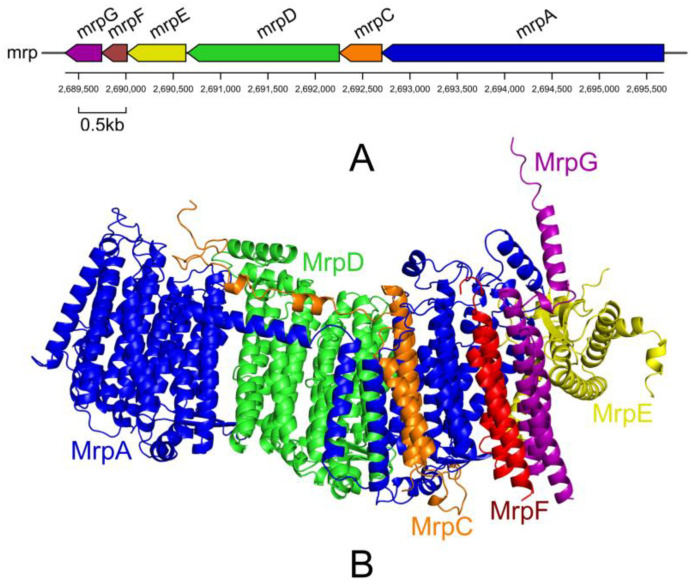
(**A**) Structure of the *mrp* operon (**B**) The predicted structure of the Mrp protein complex.

The Mrp antiporter is a heterooligomeric complex composed of multiple transmembrane proteins. It uses protons as a driving force to expel monovalent cations, thereby conferring bacterial tolerance to high Na^+^ concentrations or alkaline environments [39]. The *mrp* operon of *G. endophyticus* J2-5-19 consists of six genes, encoding an Mrp A subunit with 993 amino acid residues and five smaller subunits (Figure 6). Based on its gene structure, this operon is classified as a Group II *mrp* operon [40]. This operon is located within a predicted prophage region of the genome (Table S4, pp_22), suggesting that it may have been acquired through horizontal gene transfer.

Under salt stress conditions, halotolerant bacteria are able to maintain an osmotic pressure balance between the inside and outside of the cell by accumulating high concentrations of intracellular potassium ions. In the genome of *G. endophyticus* J2-5-19, eleven genes related to potassium ion uptake were annotated (Table S6). These genes encode potassium uptake proteins such as TrkA, KtrA/B, and KimA, as well as the two-component regulatory system proteins KdpD/KdpE [41,42,43].

**Figure 7:**
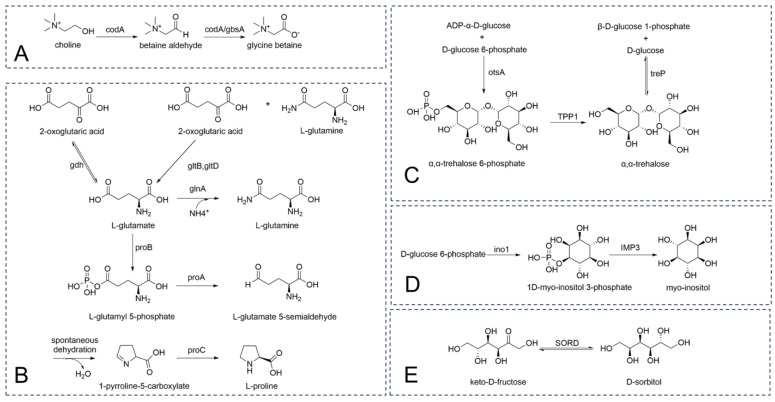
The biosynthetic mechanisms of several compatible solutes (**A**) Biosynthetic pathway of glycine betaine; (**B**) Biosynthetic pathways of glutamate, glutamine, and proline; (**C**) Biosynthetic pathway of trehalose; (**D**) Biosynthetic pathway of inositol; (**E**) Biosynthetic pathway of sorbitol.

The ion regulatory mechanism primarily functions to counteract transient salt shocks, whereas the accumulation of compatible solutes serves as a bacterial strategy to adapt to long-term osmotic stress [44]. Compatible solutes are highly water-soluble organic small molecules that help maintain high intracellular osmotic pressure without adversely affecting normal cellular physiological activities [45]. Several genes associated with the synthesis of compatible solutes were annotated in the genome of *G. endophyticus* J2-5-19, (Table S7). For example, the *codA* and *gbsA* genes are responsible for the conversion of choline to glycine betaine (Figure 7A) [46,47]. The *gdh*, *gltB/D*, *yerD*, and *glnA/E* genes are involved in the synthesis of glutamate and glutamine, with glutamate further converted into proline by the *proB/A/C* genes (Figure 7B) [48,49,50].

In addition to amino acids and their derivatives, *G. endophyticus* J2-5-19 can synthesize polyols such as trehalose, sorbitol, and inositol. The *treP*, *TPP1*, and *otsA* genes encode enzymes involved in trehalose biosynthesis (Figure 7C) [51,52,53]. The *IMP3* and *ino1* genes encode enzymes required for inositol biosynthesis (Figure 7D) [54]. Meanwhile, the *SORD* gene encodes sorbitol dehydrogenase, which catalyzes the reversible interconversion between fructose and sorbitol (Figure 7E) [55].

Multiple genes involved in the uptake of compatible solutes were identified in the genome of *G. endophyticus* J2-5-19 (Table S8). The multi-component system comprising *opuAA*, *opuAC*, and *ousW* transports glycine betaine and choline [56,57]; *proY*, *proP*, *opuE*, and *putP* are responsible for proline uptake [58,59,60]; and *gluA/B/C/D* and *glnM/Q* encode proteins involved in the transport of glutamate and glutamine, respectively [61]. The *ggtD/C/B* genes encode the GgtABCD transport system, which participates in the uptake of glucosylglycerol (GG), sucrose, and trehalose, while the *cscB* gene encodes a sucrose permease that facilitates sucrose transport into the cell [62,63]. Additionally, proteins encoded by *proP* and *lcoP* are also capable of transporting tetrahydropyrimidine [64]. The *BRA1188* and *tauB* genes are likely involved in taurine uptake [65,66].

### 3.6. Growth-Promoting Ability of the Strain and Genomic Interpretation

**Figure 8:**
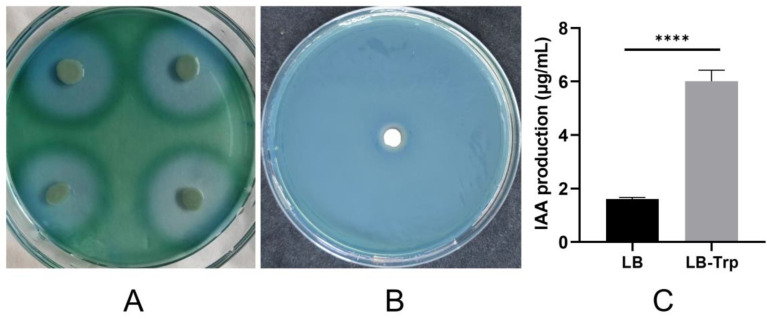
(**A**) Growth of strain J2-5-19 on casein agar plates; (**B**) Growth of strain J2-5-19 on CAS agar plates; (**C**) IAA production by strain J2-5-19 in LB medium and LB supplemented with tryptophan (5 mM). Data are presented as mean ± SD (*n* = 5 for each treatment). Statistical significance was analyzed using independent sample *t*-tests. Error bars represent the standard deviation (SD) of the mean. Asterisks indicate statistical significance (****, *p* < 0.0001).

*G. endophyticus* J2-5-19 was capable of forming a blue hydrolysis zone on casein agar plates (Figure 8A), indicating extracellular protease activity. Additionally, it grew on CAS agar plates and formed a yellow halo around the colonies (Figure 8B), demonstrating its ability to secrete siderophores. The IAA production capability of strain J2-5-19 was assessed using the Salkowski colorimetric method. In the LB medium, the IAA yield in the fermentation supernatant was 1.60 ± 0.06 μg/mL. Under supplementation with 5 mM L-Trp, the IAA yield increased to 6.01 ± 0.15 μg/mL, representing a 274% enhancement, which was statistically highly significant (*p* < 0.0001) (Figure 8C). This finding suggests that the IAA production of *G. endophyticus* J2-5-19 is tryptophan-dependent.

**Figure 9:**
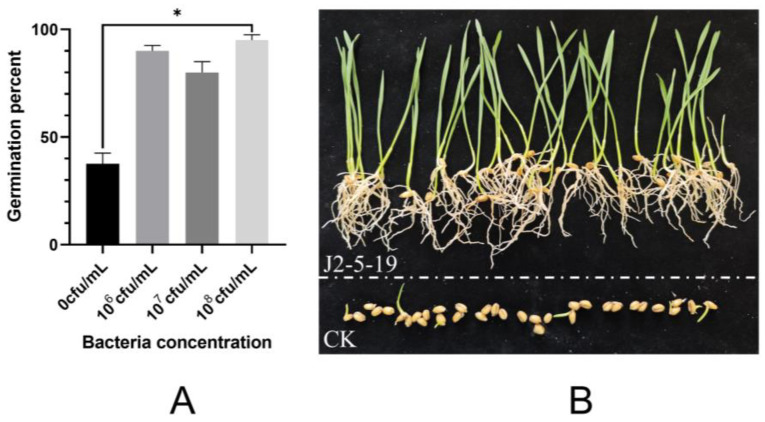
(**A**) Seed germination rates after treatment with different concentrations of bacterial suspensions under salt stress. Data are presented as median with interquartile range (*n* = 3 for each treatment). Statistical significance was determined using the Kruskal–Wallis H test with Bonferroni correction. An asterisk indicates statistical significance (*, *p* < 0.05). (**B**) Wheat seedlings treated with *G. endophyticus* J2-5-19 show improved growth compared to the control.

To investigate the growth-promoting effect of *G. endophyticus* J2-5-19 under salt stress conditions, a wheat germination experiment was conducted using a 4‰ sea salt solution to simulate a coastal saline environment. The germination rate of the control group was 37.5%. In contrast, wheat seeds treated with bacterial suspensions at concentrations of 10^6^, 10^7^, and 10^8^ cfu/mL achieved median germination rates of 90%, 80%, and 95%, respectively. The group treated with 10^8^ cfu/mL showed a significant difference relative to the control group (*p* < 0.05) (Figure 9A). Additionally, the strain significantly enhanced the growth of seedlings after germination. Over the seven-day cultivation period, coleoptiles and embryonic roots in the control group exhibited slow growth. In comparison, the seeds treated with bacterial suspension developed true leaves from coleoptiles, primary roots from embryonic roots, and secondary roots emerging from primary roots or the basal root region (Figure 9B). These results demonstrated that *G. endophyticus* J2-5-19 exerted a significant growth-promoting effect during the germination stage.

**Figure 10:**
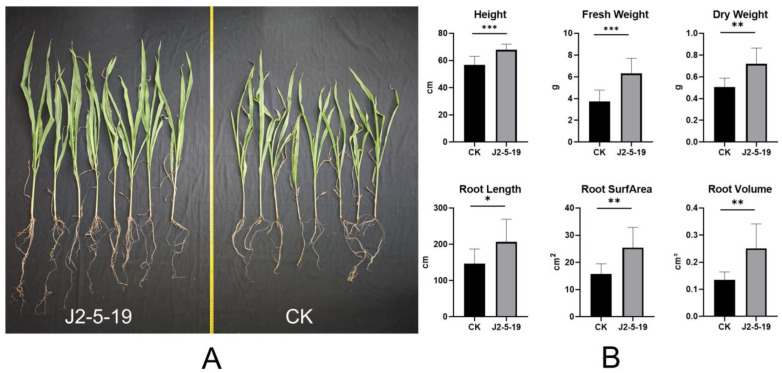
(**A**) Overall plant condition 30 days after treatment (CK: control); (**B**) Effects of strain J2-5-19 on the agronomic traits of maize. Statistical significance was analyzed using independent sample *t*-tests. Error bars represent the SD of the mean (*n* = 8 for each treatment). Asterisks indicate statistical significance (*, *p* < 0.05; **, *p* < 0.01; ***, *p* < 0.001).

A pot experiment was conducted to evaluate the plant growth-promoting effects of *G. endophyticus* J2-5-19 on maize under 4‰ soil salinity. Maize plants were harvested 30 days after treatment with *G. endophyticus* J2-5-19, and visual comparisons revealed a significant growth-promoting effect, as shown by the larger plant size and root system (Figure 10A). Compared to the control group, after 30 days of treatment with *G. endophyticus* J2-5-19 inoculum, plant height increased significantly by 19.63% (*p* < 0.001), while fresh weight and dry weight increased by 69.46% (*p* < 0.001) and 41.92% (*p* < 0.01), respectively. Total root length, root surface area, and root volume increased by 41.49% (*p* < 0.05), 61.66% (*p* < 0.01), and 87.02% (*p* < 0.01), respectively (Figure 10B). These results indicate that J2-5-19 effectively alleviated the adverse effects of salt stress on maize growth. Regarding root development, *G. endophyticus* J2-5-19 significantly promoted root development in maize seedlings, which enhanced water and nutrient absorption, thereby promoting plant growth.

Genomic annotation reveals that *G. endophyticus* J2-5-19 has the capability to degrade soil proteins and mobilize soil iron and phosphorus nutrients. The genome contains six genes encoding extracellular proteases (Table S9), which degrade proteins in the rhizosphere soil, releasing nitrogen sources and providing bioavailable amino acids for both the bacterium and the host plant. Furthermore, the genome includes multiple genes encoding alkaline phosphatases, exopolyphosphatases, and phytases (Table S10). These enzymes enable the strain to degrade organic phosphorus compounds, polyphosphates, and phytates in the soil, converting insoluble phosphates into bioavailable forms, thereby increasing the availability of phosphorus in the soil.

**Figure 11:**
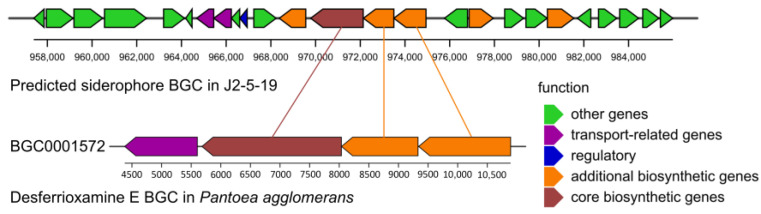
Predicted siderophore biosynthetic gene cluster (BGC) in strain J2-5-19 (**upper panel**) compared with the desferrioxamine E biosynthetic gene cluster (BGC0001572) from *Pantoea agglomerans* (**lower panel**). Lines indicate homologous regions between the two clusters.

In alkaline soils, free iron is typically scarce. Siderophores can chelate insoluble iron and increase soil bioavailable iron levels. Using antiSMASH, a gene cluster encoding a non-ribosomal peptide synthetase-independent siderophore (NRPS-independent siderophore, NIS) was predicted in the genome. MIBiG comparison showed that this gene cluster shares the highest similarity (similarity score 0.40) with the desferrioxamine E biosynthesis gene cluster in *Pantoea agglomerans* (Figure 11). Additionally, the genome contains numerous genes related to iron uptake, indicating that *G. endophyticus* J2-5-19 can competitively acquire various exogenous siderophores, including schizokinen, arthrobactin, enterobactin, petrobactin, and vulnibactin (Table S11). Furthermore, *G. endophyticus* J2-5-19 can utilize heme-bound iron, ferric citrate, and ferrous iron as iron sources.

**Figure 12:**
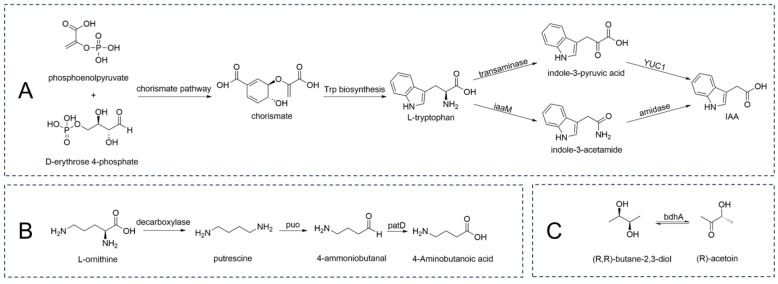
(**A**) IAA biosynthesis pathway involving tryptophan and its intermediates; (**B**) GABA biosynthesis from L-ornithine through putrescine and related intermediates; (**C**) Conversion of acetoin to (R,R)-butane-2,3-diol.

The genome of *G. endophyticus* J2-5-19 contains complete shikimate pathway-encoding genes (Table S12) and tryptophan biosynthesis pathway-encoding genes (Table S13), indicating its ability to autonomously synthesize tryptophan [67]. Additionally, the genome contains genes encoding aromatic amino acid transaminase, tryptophan-2-monooxygenase, a potential indole-3-pyruvate monooxygenase, and several amide oxidases (Table S14). This suggests that the strain may synthesize auxin using tryptophan as a precursor via two pathways. In the indole-3-pyruvate pathway, aromatic amino acid transaminase converts tryptophan into indole-3-pyruvate, which is then oxidatively decarboxylated to indole-3-acetic acid by indole-3-pyruvate flavin monooxygenase [68]. Alternatively, in the indole-3-acetamide pathway, tryptophan-2-monooxygenase oxidatively decarboxylates tryptophan to indole-3-acetamide, which is subsequently hydrolyzed by an amidohydrolase to produce indole-3-acetic acid [69] (Figure 12A).

In addition, the genome contains a gene encoding cytokinin riboside 5′-monophosphate phosphoribohydrolase (Table S15), which catalyzes the final step of cytokinin biosynthesis [70]. This suggests that *G. endophyticus* J2-5-19 has the potential to synthesize cytokinins.

*G. endophyticus* J2-5-19 contains genes associated with the synthesis of beneficial compounds, including γ-aminobutyric acid (GABA), 2,3-butanediol, acetoin, and phenazines. GABA, a key functional amino acid, enhances plant resistance to stress. Genomic analysis suggests that *G. endophyticus* J2-5-19 synthesizes GABA using putrescine and ornithine as precursors. Genes related to putrescine uptake and ornithine decarboxylation are also present in the genome (Table S16). Putrescine, produced via ornithine decarboxylation or taken up extracellularly, is converted into 4-aminobutyraldehyde by putrescine oxidase (*puo*) and subsequently oxidized to GABA by gamma-aminobutyraldehyde dehydrogenase (*patD*) [71,72] (Table S17, Figure 12B).

Volatile organic compounds, including 2,3-butanediol and acetoin, are synthesized and utilized by *G. endophyticus* J2-5-19 through the *acuC* and *bdhA* genes (Table S18). The *acuC* gene encodes an acetoin utilization protein, while *bdhA* encodes (R,R)-butanediol dehydrogenase, which catalyzes the reversible conversion between 2,3-butanediol and acetoin [73,74] (Figure 12C).

Additionally, genes encoding PhzF family phenazine biosynthesis proteins (Table S19) were identified in the genome. Phenazine compounds, known for their broad-spectrum antibacterial and antifungal activities, provide a competitive advantage to bacteria in the rhizosphere and protect plants from root pathogens.

## 4. Conclusions

In this study, a highly salt-tolerant and plant growth-promoting bacterium, *G. endophyticus* J2-5-19, was isolated and identified from the rhizosphere soil of the halophyte *Suaeda salsa* in the Yellow River Delta. *G. endophyticus* J2-5-19 demonstrates high salt tolerance, capable of growing in up to 13% NaCl, with an optimal growth range of 0% to 3%. Meanwhile, strain J2-5-19 had the ability to secrete proteases, siderophores, and indole-3-acetic acid (IAA), and significantly promoted wheat seed germination and maize growth under salt stress. Under 4‰ salt stress, the inoculation of strain J2-5-19 increased wheat germination rate from 37.5% to 95%, and significantly enhanced maize seedling height, root development, and biomass. As the first reported whole-genome of *Glutamicibacter endophyticus*, the genomic analysis revealed that strain J2-5-19 responded to transient salt stress mainly through ion transporters and adapted to long-term salt stress by synthesizing and transporting compatible solutes. Additionally, the strain harbored multiple key plant growth-promoting genes, such as those involved in IAA biosynthesis, siderophore synthesis, and GABA synthesis. As a novel salt-tolerant plant growth-promoting bacterium, *G. endophyticus* J2-5-19 contributes to the enrichment of microbial resources and holds significant potential for improving saline-alkaline agricultural ecosystems and increasing crop yields under salt stress conditions.

## Figures and Tables

**Table 2:** Differential biochemical characteristics of strain J2-5-19 and strain EGI 6500322T. +, positive; −, negative; w, weak positive; ND, not determined.

Characteristics	J2-5-19	EGI 6500322T
VP test	−	ND
Hydrolysis of		
Tween 60	−	+
Urea	−	+
PNPG	+	−
Assimilation of		
Mannose	w	+
Mannitol	−	+

**Table 3:** Results of Average Nucleotide Identity (ANI) calculations, digital DNA–DNA hybridization (dDDH) estimates, and GC content differences between closely related strains.

	J2-5-19 & *G. endophyticus*	*G. ardleyensis* & *G. bergerei*
ANIb	95.93%	96.84%
ANIm	96.26%	97.96%
DDH estimate	67.4% (64.4–70.3%)	79.7% (76.8–82.4%)
Probability that DDH > 70%	73.15%	90.36%
Difference in GC%	0.01	0.24

## Data Availability

Data are contained within the article or Supplementary Materials. The sequencing data generated in this study have been uploaded to the NCBI database. The GenBank accession number for the 16S rRNA gene is PQ606648, and the genome sequencing and assembly data are available under BioProject PRJNA1187829.

## Supplementary Materials

The following supporting information can be downloaded at: https://www.mdpi.com/article/10.3390/microorganisms13010208/s1.
