# Supplementary material for: Association Analysis of the Genomic and Functional Characteristics of Halotolerant Glutamicibacter endophyticus J2-5-19 from the Rhizosphere of Suaeda salsa
Source: Microorganisms. 2025 Jan 18;13(1):208. doi: 10.3390/microorganisms13010208 (PMC11767460; doi:10.3390/microorganisms13010208)
Supplement: Supplementary file 1 [file microorganisms-13-00208-s001.zip › microorganisms-3419309-supplementary.pdf]

Association Analysis of the Genomic and Functional Characteristics of Halotlerant *Glutamicibacter endophyticus* J2-5-19 from the Rhizosphere of *Suaeda salsa*

Longhao Sun[1,2] , Shanshan Sun[1,2],TianYang Liu[1,2], XinMin Lei [1,2], Ruiqi Liu[1,2], JunYi Zhang[1,2], ShanShan Dai[1,2],Jing Li[1,2] and Yanqin Ding [1,2,]*

1 Department of Microbiology, College of Life Sciences, Shandong Agricultural University, Taian 271018, China; sdaulty@163.com (T.L.); lrq13165462355@163.com (R.L.)
2 Shandong Engineering Research Center of Plant-Microbial Restoration for Saline-Alkali Land, Taian 271018, China
* Correspondence: dyq@sdau.edu.cn; Tel./Fax: +86-538-824265

Table S1. Genomes of type strains used for comparative genomic analysis.

**Type strains**	**RefSeq Accession**	**Size(bp)**	**Contigs**
Glutamicibacter ardleyensis CGMCC 1.3685	GCF_014644555.1	3,936,280	28
Glutamicibacter arilaitensis Re117	GCF_000197735.1	3,918,192	3
Glutamicibacter bergerei JCM 13567	GCF_039534605.1	3,911,806	127
Glutamicibacter creatinolyticus JCM 10102	GCF_039534605.1	3,465,207	44
Glutamicibacter endophyticus JCM 30091	GCM10027417	3,455,460	19
Glutamicibacter halophytocola KLBMP 5180	GCF_039534605.1	3,918,424	2
Glutamicibacter mishrai S5-52T	GCF_012221945.1	3,570,747	1
Glutamicibacter mysorens DSM 12798	GCF_012221945.1	3,459,735	1
Glutamicibacter nicotianae NBRC 14234	GCF_012221945.1	3,554,887	43
Glutamicibacter protophormiae DSM 20168	GCF_017876615.1	3,872,967	2
Glutamicibacter uratoxydans NBRC 15515	GCF_017876615.1	3,786,014	49

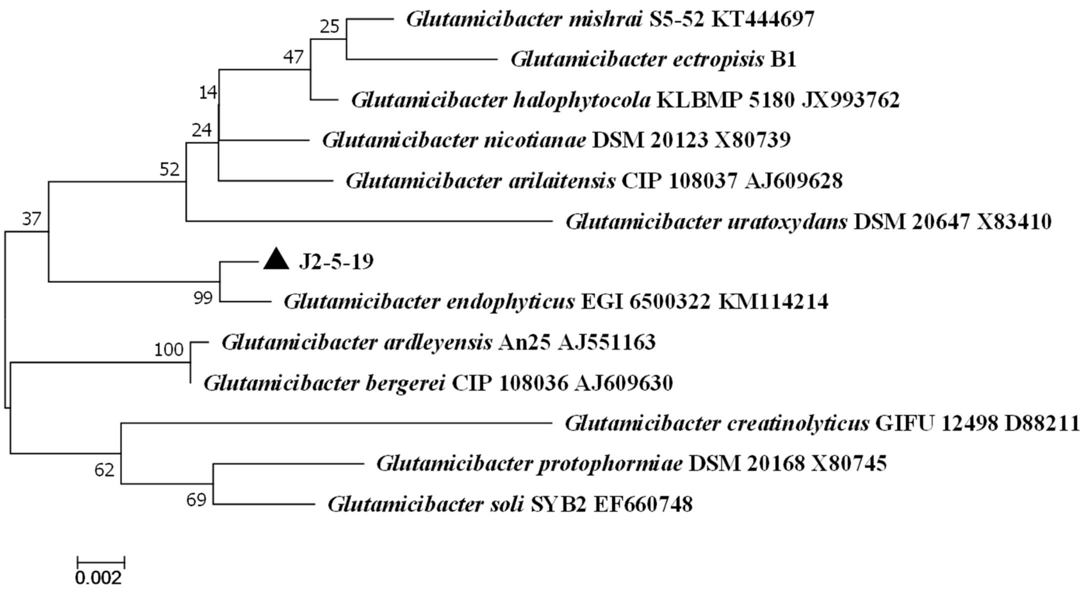

Figure S1. Maximum Likelihood phylogenetic tree based on 16S rRNA gene sequences of strain J2-5-19 and representative *Glutamicibacter* species. Bootstrap values at each branch node indicate. the support percentages from 1000 replicates.

Table S2. CRISPR-Cas prediction results

SeqID	Start	End	Number of spacers	Length
chr	2231685	2231779	1	95
chr	2644105	2644188	1	84

Table S3. Overview of the distribution of genomic islands present in the genome

IsID	start	end	gene
Is1	1033056	1053594	16
Is2	1034580	1056429	15
Is3	110841	124901	8
Is4	1190441	1196473	2
Is5	1247646	1286868	38
Is6	1261935	1293981	39
Is7	1408881	1415138	7
Is8	1507087	1512181	5
Is9	1515487	1523728	4
Is10	1525272	1539189	5
Is11	1700410	1705180	7
Is12	1725707	1732761	4
Is13	173829	191505	22
Is14	1917324	1923178	7
Is15	1963674	1969091	4
Is16	2035630	2039882	6
Is17	204083	210751	11
Is18	2422149	2429927	7
Is19	2787641	2795843	8
Is20	2839235	2846531	9
Is21	3215886	3230254	10
Is22	3338961	3343259	4
Is23	515622	525510	3
Is24	663575	670813	8
Is25	855186	862247	12
Is26	99301	110975	6

Table S4. Summary of prophage prediction results

Prophage number	Start	Stop
pp_1	145852	151567
pp_2	177919	183719
pp_3	376805	396664
pp_4	456300	485043
pp_5	561739	602685
pp_6	667299	710880
pp_7	794904	823882
pp_8	1064722	1080049
pp_9	1174567	1205504
pp_10	1291398	1305606
pp_11	1330572	1339758
pp_12	1420437	1442299
pp_13	1580203	1643879
pp_14	1654464	1683303
pp_15	1748409	1779549
pp_16	1847962	1851710
pp_17	2128423	2145868
pp_18	2290067	2297219
pp_19	2341201	2360197
pp_20	2378372	2392935
pp_21	2407884	2431840
pp_22	2684415	2762648
pp_23	3001366	3028052
pp_24	3160227	3246142
pp_25	3370726	3383480

Table S5. Genes encoding cation/proton antiporters

Gene ID	Start	End	Strand	Name	Subject ID	Probable Function
chr_350	354554	356134	-	*apnhaP*	Q93HU4	Na^{+}/H^{+} antiporter ApNhaP
chr_775	828755	829999	-	*nhaA*	A0JR38	Na^{+}/H^{+} antiporter NhaA
chr_2446	2689358	2689744	-	*mrpG*	Q9RGY9	Na^{+}/H^{+} antiporter subunit G
chr_2447	2689744	2690013	-	-	WP_022874321.1	monovalent cation/H^{+} antiporter complex subunit F
chr_2448	2690010	2690633	-	-	WP_204675441.1	Na^{+}/H^{+} antiporter subunit E
chr_2449	2690650	2692254	-	*mrpD*	Q9RGZ2	Na^{+}/H^{+} antiporter subunit D
chr_2450	2692254	2692703	-	*mrpC*	Q9RGZ3	Na^{+}/H^{+} antiporter subunit C
chr_2451	2692700	2695681	-	-	WP_188948700.1	Na^{+}/H^{+} antiporter subunit A
chr_2666	2932877	2934082	-	gerT	B3VQ24	Probable Na^{+}/H^{+} antiporter GerT

Table S6. Genes encoding proteins related to potassium ion uptake

Gene ID	Start	End	Strand	Name	Subject ID	Probable Function
chr_410	422191	423495	+	*ktrB*	O32081	Ktr system potassium uptake protein B
chr_411	423563	425407	+	*kdpD*	P9WGL2	Sensor protein KdpD
chr_412	425404	426087	+	*kdpE*	P9WGN1	Transcriptional regulatory protein KdpE
chr_1967	2171613	2173574	-	*kimA*	P96589	Potassium transporter KimA
chr_1968	2173755	2174432	+	*trkA*	Q53949	Trk system potassium uptake protein TrkA
chr_1969	2174443	2175108	+	*trkA*	P9WFZ3	Trk system potassium uptake protein TrkA
chr_1970	2175114	2175824	-	-	PRB72160.1	potassium ABC transporter
chr_2370	2599623	2601053	+	*ktrB*	O32081	Ktr system potassium uptake protein B
chr_2371	2601115	2601717	+	*ktrA*	O32080	Ktr system potassium uptake protein A
chr_2914	3189229	3189870	-	*ktrA*	O87952	Ktr system potassium uptake protein A
chr_2915	3189920	3191290	-	*ktrB*	O32081	Ktr system potassium uptake protein B

Table S7. Genes related to the synthesis of compatible solutes

Gene ID	Start	End	Strand	Name	Subject ID	Probable Function
chr_657	705477	707051	+	*codA*	Q7X2H8	Choline oxidase
chr_656	703935	705458	+	*gbsA*	P71016	Betaine aldehyde dehydrogenase
chr_2367	2596229	2597572	-	*gdh*	Q8RQP4	NADP-specific glutamate dehydrogenase
chr_1915	2114878	2116308	-	*gltD*	P9WN19	Glutamate synthase [NADPH] small chain
chr_1916	2116328	2120968	-	*gltB*	P96218	Glutamate synthase [NADPH] large chain
chr_2590	2847755	2849404	-	*yerD*	O34849	Glutamate synthase large subunit-like protein YerD
chr_2003	2214546	2215970	+	*glnA*	P15106	Glutamine synthetase
chr_2005	2216540	2219566	-	*glnE*	Q6AFH2	Bifunctional glutamine synthetase adenylyltransferase/adenylyl-removing enzyme
chr_1332	1437853	1438872	+	*SORD*	P0DMQ6	Sorbitol dehydrogenase
chr_1798	1972809	1973954	-	*proB*	Q47MV7	Glutamate 5-kinase
chr_1797	1971490	1972749	-	*proA*	Q6AFX9	Gamma-glutamyl phosphate reductase
chr_2373	2602695	2603537	-	*proC*	P22008	Pyrroline-5-carboxylate reductase
chr_48	45242	48439	+	*treP*	Q8L164	α,α-trehalose phosphorylase
chr_495	534835	535614	-	*TPP1*	Q75WV3	Probable trehalose-phosphate phosphatase 1
chr_496	535611	537092	-	*otsA*	A0R4M9	Trehalose-6-phosphate synthase
chr_2893	3167502	3168317	-	*IMP3*	P54928	Inositol monophosphatase 3
chr_3191	3496349	3497434	+	*ino1*	A0R7G6	Inositol-3-phosphate synthase

Table S8. Genes related to the uptake of compatible solutes

Gene ID	Start	End	Strand	Name	Subject ID	Probable Function
chr_299	291317	292774	+	*proP*	Q79VC4	Ectoine/proline transporter ProP
chr_1261	1362213	1364099	+	*lcoP*	Q8NN75	Betaine/ectoine transporter LcoP
chr_1497	1654464	1655354	-	*opuAC*	P46922	Glycine betaine-binding protein OpuAC
chr_1498	1655405	1656301	-	*ousW*	E0SCY2	Glycine betaine/choline transport system permease protein OusW
chr_1499	1656305	1657570	-	*opuAA*	P46920	Glycine betaine transport ATP-binding protein OpuAA
chr_2115	2349993	2350763	+	*gluA*	P48243	Glutamate transport ATP-binding protein GluA
chr_2116	2350800	2351633	+	*gluB*	P48242	Glutamate-binding protein GluB
chr_2117	2351737	2352417	+	*gluC*	Q8RQL5	Glutamate transport system permease protein GluC
chr_2118	2352414	2353277	+	*gluD*	Q8RQL4	Glutamate transport system permease protein GluD
chr_2316	2546565	2547359	+	*glnM*	O34671	Probable glutamine ABC transporter permease protein GlnM
chr_2317	2547372	2548160	+	*glnQ*	P27675	Glutamine transport ATP-binding protein GlnQ
chr_383	390910	392316	-	*proY*	P37460	Proline-specific permease ProY
chr_532	575586	577100	-	*opuE*	O06493	Osmoregulated proline transporter OpuE
chr_2603	2865619	2867124	+	*putP*	P94392	High-affinity proline transporter PutP
chr_2978	3264615	3266165	-	*opuE*	O06493	Osmoregulated proline transporter OpuE
chr_3030	3327213	3328664	-	*proY*	P37460	Proline-specific permease ProY
chr_327	325202	326149	-	*ggtD*	Q55473	Osmoprotective compounds uptake permease protein GgtD
chr_328	326146	327285	-	*ggtC*	Q55472	Osmoprotective compounds uptake permease protein GgtC
chr_329	327355	328695	-	*ggtB*	Q55471	Osmoprotective compounds-binding protein GgtB
chr_233	224284	225567	-	*cscB*	P30000	Sucrose permease
chr_1821	2002453	2003217	-	*BRA1188*	Q8FUN2	Probable ABC transporter permease protein
chr_1822	2003297	2004028	-	*tauB*	Q1M7R4	Taurine import ATP-binding protein TauB

Table S9. Genes encoding exoproteases

Gene ID	Start	End	Strand	Name	Subject ID	Probable Function
chr_415	429087	432353	-	*vpr*	P29141	Minor extracellular protease Vpr
chr_674	724392	725912	+	*apr*	P00781	Subtilisin DY
chr_2069	2293449	2294870	-	*pepD*	O53896	Serine protease PepD
chr_2396	2626944	2628134	-	*Rv3671c*	P9WHR9	Serine protease Rv3671c
chr_2629	2895412	2896557	+	*BH0855*	P41363	Thermostable alkaline protease
chr_2995	3289214	3291175	+	*XCC0851*	P23314	Extracellular protease

Table S10. Genes encoding phosphatases

Gene ID	Start	End	Strand	Name	Subject ID	Probable Function
chr_362	367393	368967	+	*phoD*	P42251	Alkaline phosphatase D
chr_673	723381	724325	+	*ppx2*	P96374	Exopolyphosphatase 2
chr_2275	2506689	2508905	+	*ppk*	Q9KZV6	Polyphosphate kinase
chr_2318	2548221	2550128	-	-	WP_141362385.1	PhoX family phosphatase
chr_2321	2552596	2554686	-	-	WP_060616998.1	PhoX family phosphatase
chr_2375	2604417	2605436	-	*SCO3348*	WP_141362282.1	Ppx/GppA family phosphatase
chr_2627	2892851	2894305	+	*phoA*	P35483	Alkaline phosphatase H
chr_2887	3160227	3162755	-	-	WP_096284931.1	esterase-like activity of phytase family protein

Table S11. Genes Related to Iron and Siderophore Uptake

Gene ID	Start	End	Strand	Name	Subject ID	Probable Function
chr_1475	1632956	1633957	-	*yfiY*	O31567	Probable siderophore-binding lipoprotein YfiY
chr_1476	1634062	1635096	+	*fepD*	P23876	Ferric enterobactin transport system permease protein FepD
chr_1477	1635093	1636112	+	*yfhA*	O31569	Probable siderophore transport system permease protein YfhA
chr_1482	1640567	1641814	-	*efeN*	P39597	Deferrochelatase
chr_1484	1643040	1643879	-	*efeU*	Q8FJ36	Ferrous iron permease EfeU
chr_2537	2789928	2791877	-	*yusV*	O32188	Probable siderophore transport system ATP-binding protein YusV
chr_2538	2791880	2792917	-	*yfhA*	O31569	Probable siderophore transport system permease protein YfhA
chr_2539	2792914	2793930	-	*yfiZ*	O31568	Probable siderophore transport system permease protein YfiZ
chr_2541	2794797	2795843	-	*yfmC*	O34348	Fe(3+)-citrate-binding protein YfmC
chr_2877	3148047	3148856	-	*fepC*	P23878	Ferric enterobactin transport ATP-binding protein FepC
chr_2878	3148853	3149920	-	*fepG*	P23877	Ferric enterobactin transport system permease protein FepG
chr_2879	3149917	3150918	-	*fepD*	P23876	Ferric enterobactin transport system permease protein FepD
chr_2880	3150950	3151888	-	*yfiY*	O31567	Probable siderophore-binding lipoprotein YfiY
chr_2881	3151927	3152778	-	*viuB*	Q56743	Vulnibactin utilization protein ViuB
chr_2897	3171490	3173328	-	*fbpB*	Q44123	Ferric transport system permease protein FbpB
chr_2985	3270995	3271918	-	*efeB*	Q66BP4	Deferrochelatase
chr_3049	3349033	3350019	+	*yclQ*	P94421	Petrobactin-binding protein YclQ
chr_3050	3350119	3351099	+	*fatD*	Q81XB1	Petrobactin import system permease protein FatD
chr_3051	3351194	3352144	+	*fatC*	Q81XB2	Petrobactin import system permease protein FatC
chr_3052	3352141	3352896	+	*fatE*	Q81XB3	Petrobactin import ATP-binding protein FatE
chr_3119	3418703	3419476	+	*yusV*	O32188	Probable siderophore transport system ATP-binding protein YusV

Table S12. Genes encoding chorismate pathway enzymes

Gene ID	Start	End	Strand	Name	Subject ID	Probable Function
chr_2486	2730772	2731887	-	*aroG*	P35170	Phospho-2-dehydro-3-deoxyheptonate aldolase
chr_1150	1242881	1244272	+	*aroH*	P80574	Phospho-2-dehydro-3-deoxyheptonate aldolase
chr_1694	1862979	1864070	-	*aroB*	B8H8V5	3-dehydroquinate synthase
chr_2395	2626486	2626947	+	*aroQ*	A3PP56	3-dehydroquinate dehydratase
chr_1697	1865857	1866687	-	*ARO1*	Q6C1X5	Pentafunctional AROM polypeptide
chr_1695	1864133	1864627	-	*aroK*	A5GQN5	Shikimate kinase
chr_1209	1302224	1303552	-	*aroA*	B2GLU6	3-phosphoshikimate 1-carboxyvinyltransferase
chr_1696	1864628	1865827	-	*aroC*	A1R705	Chorismate synthase

Table S13. Genes encoding tryptophan biosynthesis

Gene ID	Start	End	Strand	Name	Subject ID	Probable Function
chr_1923	2126266	2127849	-	*trpE*	P96556	Anthranilate synthase component 1
chr_24	21943	22587	+	*trpG*	P9WN35	Anthranilate synthase component 2
chr_1634	1796936	1797952	-	*trpD*	A0JX18	Anthranilate phosphoribosyltransferase
chr_1920	2124460	2125275	-	*trpC*	A0JVL1	Indole-3-glycerol phosphate synthase
chr_2593	2853129	2854697	+	*trpF*	Q71Z39	N-(5'-phosphoribosyl)anthranilate isomerase
chr_1919	2123158	2124411	-	*trpB*	A5CRV6	Tryptophan synthase beta chain
chr_1918	2122376	2123158	-	*trpA*	Q6AF66	Tryptophan synthase alpha chain

Table S14. Genes encoding IAA biosynthesis

Gene ID	Start	End	Strand	Name	Subject ID	Probable Function
chr_2749	3009202	3010449	+	*OCC_04335*	H3ZPL1	Aromatic-amino-acid aminotransferase 1
chr_2969	3254963	3256198	+	*OCC_04335*	H3ZPL1	Aromatic-amino-acid aminotransferase 1
chr_980	1060599	1062389	+	*YUC1*	Q9SZY8	Probable indole-3-pyruvate monooxygenase YUCCA1
chr_2883	3154352	3156061	-	*iaaM*	P25017	Tryptophan 2-monooxygenase
chr_2954	3237891	3239225	+	*YJL213W*	QDY67635.1	Amidohydrolase family protein
chr_885	952705	954327	+	*ytcJ*	O34355	Putative amidohydrolase YtcJ
chr_2738	2999720	3001354	-	*ytcJ*	O34356	Putative amidohydrolase YtcJ
chr_3159	3456541	3458163	+	*ytcJ*	O34355	Putative amidohydrolase YtcJ

Table S15. Genes encoding cytokinin biosynthesis

Gene ID	Start	End	Strand	Name	Subject ID	Probable Function
chr_603	654727	655278	+	*PA4923*	P48636	Cytokinin riboside 5'-monophosphate phosphoribohydrolase

Table S16. Genes encoding ornithine decarboxylase and putrescine uptake

Gene ID	Start	End	Strand	Name	Subject ID	Probable Function
chr_2647	2911312	2913882	-	-	PRA10690.1	ornithine decarboxylase
chr_761	814048	815529	-	*aguD*	Q9CEY5	Probable agmatine/putrescine antiporter AguD
chr_884	951249	952658	+	*puuP*	P76037	Putrescine importer PuuP
chr_1019	1099581	1100417	+	*potC*	P45169	Spermidine/putrescine transport system permease protein PotC
chr_2480	2724285	2725085	-	*potC*	Q83RR7	Spermidine/putrescine transport system permease protein PotC
chr_2481	2725095	2726012	-	*potB*	P0CL49	Spermidine/putrescine transport system permease protein PotB
chr_2482	2726024	2727184	-	*potA*	Q47T99	Spermidine/putrescine import ATP-binding protein PotA
chr_2483	2727306	2728541	-	*potD-B*	P45168	Spermidine/putrescine-binding periplasmic protein 1
chr_2763	3022783	3024138	+	*puuP*	P76037	Putrescine importer PuuP
chr_2855	3126862	3128355	+	*puuP*	P76037	Putrescine importer PuuP

Table S17. Genes encoding GABA biosynthesis

Gene ID	Start	End	Strand	Name	Subject ID	Probable Function
chr_2686	2950119	2951561	-	*puo*	P40974	Putrescine oxidase
chr_2514	2763026	2764516	+	*patD*	A9MQY3	Gamma-aminobutyraldehyde dehydrogenase

Table S18. Genes related to acetoin and 2,3-butanediol

Gene ID	Start	End	Strand	Name	Subject ID	Probable Function
chr_1356	1466164	1467225	+	*bdhA*	O34788	(R,R)-butanediol dehydrogenase
chr_2369	2598280	2599485	-	*acuC*	P39067	Acetoin utilization protein AcuC

Table S19. Genes encoding phenazine biosynthesis

Gene ID	Start	End	Strand	Name	Subject ID	Probable Function
chr_245	235068	235907	-	-	WP_243228530.1	PhzF family phenazine biosynthesis protein
chr_1301	1407603	1408334	-	-	WP_068734006.1	PhzF family phenazine biosynthesis protein
chr_2548	2800990	2801997	-	-	WP_096283215.1	PhzF family phenazine biosynthesis protein
